# Diagnosing hereditary cancer predisposition in men with prostate cancer

**DOI:** 10.1038/s41436-020-0830-5

**Published:** 2020-05-22

**Authors:** Mary Pritzlaff, Yuan Tian, Patrick Reineke, A. J. Stuenkel, Kyle Allen, Stephanie Gutierrez, Michelle Jackson, Jill S. Dolinsky, Holly LaDuca, Jianfeng Xu, Mary Helen Black, Brian T. Helfand

**Affiliations:** 1 Ambry Genetics, Aliso Viejo, CA USA; 2 Division of Urology, Northshore University Health System, Evanston, IL USA

**Keywords:** genetic testing, prostate cancer, multigene panel testing, HRD, MMRD

## Abstract

**Purpose:**

We describe the pathogenic variant spectrum and identify predictors of positive results among men referred for clinical genetic testing for prostate cancer.

**Methods:**

One thousand eight hundred twelve men with prostate cancer underwent clinical multigene panel testing between April 2012 and September 2017. Stepwise logistic regression determined the most reliable predictors of positive results among clinical variables reported on test requisition forms.

**Results:**

A yield of 9.4–12.1% was observed among men with no prior genetic testing. In this group, the positive rate of *BRCA1* and *BRCA2* was 4.6%; the positive rate for the mismatch repair genes was 2.8%. Increasing Gleason score (odds ratio [OR] 1.19; 95% confidence interval [CI] 0.97–1.45); personal history of breast or pancreatic cancer (OR 3.62; 95% CI 1.37–9.46); family history of breast, ovarian, or pancreatic cancer (OR 2.32 95% CI 1.48–3.65); and family history of Lynch syndrome–associated cancers (OR 1.97; 95% CI 1.23–3.15) were predictors of positive results.

**Conclusion:**

These results support multigene panel testing as the primary genetic testing approach for hereditary prostate cancer and are supportive of recommendations for consideration of germline testing in men with prostate cancer. Expanding the criteria for genetic testing should be considered as many pathogenic variants are actionable for treatment of advanced prostate cancer.

## INTRODUCTION

Germline pathogenic variants (PVs) in cancer predisposition genes are reported in 7.3% to 11.8% of aggressive prostate cancer (PC) cases, including genes associated with homologous repair deficiency (HRD) (e.g., *BRCA1*, *BRCA2*, *ATM*, *BRIP1*, *CHEK2*, *NBN*, *BARD1*, *RAD51C*, *MRE11A*, and *PALB2)*, and mismatch repair (MMR) deficiency (e.g., *MLH1*, *MSH2*, *MSH6*, and *PMS2)*.^1,2^ Most of these genes have clear management guidelines for early cancer detection and risk reduction, which may benefit the patient and family members. Their relationship to PC screening and management has garnered recent interest.^3^ Previous reports suggest that PVs in *BRCA1/2* confer increased risk for PC associated with poor survival and younger age at diagnosis; *HOXB13* PVs also are associated with a young age at diagnosis.^4,5^ Men at increased risk for aggressive or earlier-onset disease may choose more aggressive screening or earlier intervention.^6^ Men with HRD or MMR-deficient metastatic prostate tumors may also benefit from targeted therapeutics, such as pembrolizumab, platinum therapies, or PARP inhibitors.^7–9^

Despite improved understanding of the prevalence of PVs among men with PC, it remains unclear which men will most benefit from genetic testing. Historically, the Hopkins criteria, i.e., ≥3 affected first-degree relatives, affected relatives in three successive generations, or ≥2 relatives affected at 55 years or younger, provided a working definition of hereditary PC (HPC), but there is little evidence that HPC is associated with DNA repair gene variants. Recent data suggest that aggressive disease or family history of other cancers may be a better predictor for germline PVs in men with PC.^1,2^ In 2017, expert consensus guidelines recommended genetic testing for men with HPC; men with ≥2 close unilinear relatives with a cancer associated with hereditary breast and ovarian cancer (HBOC) or Lynch syndrome (LS), men with metastatic castration-resistant PC, and men with somatic (PVs) identified via tumor testing.^10^ However, these testing guidelines are based on limited evidence. Thus, there is an urgent need to determine a more robust way of identifying men with PVs so they may benefit from early screening/intervention and therapeutic options. This study evaluates predictors of germline PV status in a large cohort of men with a personal history of PC who underwent clinical genetic testing to help inform clinical testing guidelines.

## MATERIALS AND METHODS

### Study population

Study participants included men with PC who underwent hereditary cancer multigene panel testing (MGPT) between April 2012 and September 2017 at a clinical diagnostic laboratory (Ambry Genetics) (*n* = 1878). Men with a *BRCA1/2* PV reported in their family prior to testing were excluded (*n* = 66), leaving a total of 1812 individuals in the analyzed cohort. Patients who had prior genetic screening or testing (*N* = 150), including *BRCA1/2* or LS germline testing, immunohistochemical screening for MMR deficiency in tumors, and other somatic testing, were analyzed separately to minimize bias in PV detection rates; 1662 men had no prior testing. Demographic and clinical data including age at testing, ethnicity, age of diagnosis, Gleason score, metastatic status, and personal and family history of cancer were collected through retrospective review of test requisition forms and other clinical documentation (e.g., pedigrees and consult notes) provided to the laboratory.

### Laboratory methods

Depending on the type of clinical tests ordered, men underwent analysis of up to 67 cancer susceptibility genes. The frequencies of each gene tested are described in Table S1. Sanger or next-generation sequencing analysis was performed for all coding domains and well into the flanking 5’ and 3’ ends of all introns and untranslated regions, along with gross deletion/duplication analysis of covered exons and untranslated regions. Exceptions included *GREM1*, *EPCAM*, and *MITF*, for which analysis was limited to alterations known to be associated with disease (Table S1).

### Variant classification

Variants were interpreted using a five-tier variant classification protocol (pathogenic variant [PV]; variant, likely pathogenic [VLP]; variant of unknown significance [VUS]; variant, likely benign [VLB]; and benign), based on published recommendations from the American College of Medical Genetics and Genomics and the International Agency for Research on Cancer.^11–13^ All identified alterations were deposited in ClinVar.

### Statistical analysis

Descriptive statistics for the PC cohort are summarized as median (interquartile range [IQR]) for continuous and percentages for categorical characteristics. Logistic regression estimated odds ratios (OR) (95% confidence interval [CI]) for univariate associations between PV carrier status and personal and family history characteristics, by gene and for the combined set of PC predisposition genes (*ATM*, *BRCA1*, *BRCA2*, *CHEK2*, *EPCAM*, *HOXB13*, *MLH1*, *MSH2*, *MSH6*, *NBN*, *PALB2*, *PMS2*, *RAD51D*, and *TP53*). Stepwise logistic regression determined the most informative set of predictors from the following: continuous age at PC diagnosis; Ashkenazi Jewish ethnicity; personal history of non-PC; personal history of breast or pancreatic cancer; family history (presence of first- or second-degree relative) of cancer; >1 first or second-degree relative with PC; >1 first- or second-degree relative with breast, ovarian, or pancreatic cancer; >1 first- or second-degree relative with LS-related cancer (colorectal, endometrial, gastric, ovarian, pancreatic, small bowel, urothelial, kidney, or bile duct cancer); and Gleason score. The stepwise selection procedure aimed to minimize Akaike’s information criterion (AIC), allowing a maximum of 1000 backward and forward steps. All analyses were conducted in R V3.3.3.

## RESULTS

### Demographics

The study cohort was primarily Caucasian (70%; Table 1). The median age at testing was 66 (IQR 59, 73) years and the median age of PC diagnosis was 60 (IQR 54, 66) years. Forty-two percent had a personal history of other cancers, including colorectal (11.1%), breast (5.8%), and pancreatic (6.1%). Most individuals (92.4%) had a family history of cancer in at least one close (first, second, or third degree) relative; with 52.0% having a history of breast cancer and 50.6% PC. Thirty-six percent of men had more than one relative with breast, ovarian, or pancreatic cancer and 31.6% had more than one family member with a LS-related cancer. Gleason scores were available for approximately half of PC cases. Of the men with available Gleason scores, 17.7% were ≤6; 43.2% were equal to 7; and 39.1% were 8–10.

**Table 1:** Descriptive characteristics of the entire cohort (*N* = 1812)^d^.

	Total (*N*)	Total (%)
Age at testing (IQR)	66 (59,73)	
Race/ethnicity		
African American/Black	109	6.0%
Ashkenazi Jewish	165	9.1%
Asian	23	1.3%
Caucasian	1268	70.0%
Hispanic	47	2.6%
Middle Eastern	7	0.4%
Mixed ethnicity	65	3.6%
Native American	2	0.1%
Other	8	0.4%
Unknown	118	6.5%
Personal history of other cancers^a^		
Yes	758	41.8%
No	1054	58.2%
Personal history of other cancers^c^		
Breast	105	5.8%
Colorectal	201	11.1%
Pancreatic	111	6.1%
Other	487	26.9%
None	1054	58.2%
Family history of cancer^b,c^		
Yes	1674	92.4%
No	138	7.6%
Family history of cancer		
Breast	943	52.0%
Colorectal	578	31.9%
Pancreatic	313	17.3%
Prostate	917	50.6%
Other	1177	65.0%
Gleason score		
Low (<7)	162	17.7%
Intermediate (7)	396	43.2%
High (8–10)	359	39.1%
Missing	895	
>1 Family member with breast, ovarian, or pancreatic cancer		
Yes	649	35.8%
No	1163	64.2%
>1 Family member with Lynch syndrome–related cancer		
Yes	572	31.6%
No	1240	68.4%
Test ordered		
ProstateNext	284	15.7%
Other multigene panel tests	1528	84.3%

^a^Cancer status “not provided” and “no” are treated as the same.

^b^Only first-, second-, and third-degree relatives are included.

^c^Nonmelanoma skin cancers and unspecified cancers are excluded.

^d^Excluded 66 probands with a known *BRCA1/2* pathogenic variant in the family.

### Germline genetic test results

**Fig. 1:**
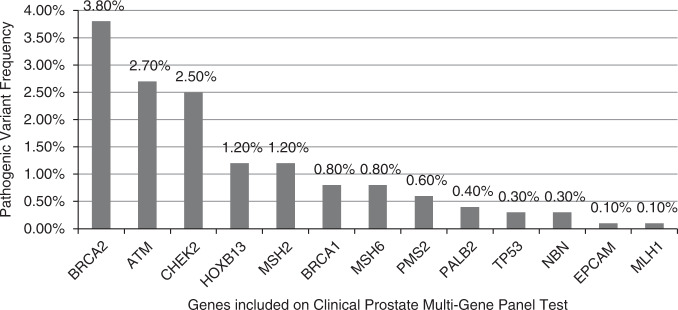
Pathogenic variant frequencies in genes associated with prostate cancer among men with no prior testing. Includes men with any test ordered (*BRCA1/2*, ProstateNext, or other multigene panel test) with no prior genetic testing.

Among men with no prior genetic testing, the yield of a 14-gene hereditary prostate cancer panel ProstateNext was 9.4%, with 26/277 testing positive for a PV. The yield of all other MGPTs combined was 12.1%, with 168/1385 testing positive (Table S2). Among candidate prostate cancer genes, PV frequencies were highest for *BRCA2* (3.8%), *ATM* (2.7%), and *CHEK2* (2.5%). PVs were also seen in *MSH2*, *HOXB13*, *BRCA1*, *MSH6*, *PMS2*, *PALB2*, *TP53*, *NBN*, *MLH1*, and *EPCAM*. No PVs were detected in *RAD51D* (Fig. 1, Table S3). Among all men with no prior genetic testing, the pooled frequency of PV in therapeutically actionable genes (*BRCA1/2* and MMR genes) was 7.4%. Nine men who underwent MGPT (0.6%) had PVs in genes not currently associated with PC; all had clinical history consistent with the alteration identified (Table S4). Fourteen men in the entire cohort (0.7%) were found to have more than one PV (Table S5). Among men with prior genetic testing, results for 34/40 were consistent with previous findings, such as confirmation of somatic PVs in the germline or PVs that were concordant with tumor immunohistochemistry (IHC) results; 6/40 were found to have discordant results with tumor testing or additional PVs identified on expanded testing that were not detected with initial limited testing (Figure S1).

### Predictors of positive test results

#### Univariate analysis

The type of panel (ProstateNext vs. all other panels) used for testing was not associated with positive or negative results (*p* = 0.10). There was no significant difference in median (IQR) age at PC diagnosis for men testing positive versus negative (59 [11] vs. 60 [12] years; *p* = 0.32) (Table 2). Having a personal history of another cancer in addition to PC was significantly associated with a positive test result, (OR 1.35; 95% CI 1.03–1.75; *p* = 0.027). Having a first-degree relative with PC was not significant (*p* = 0.59), nor was having any family history of PC (*p* = 0.56). Similarly, men who satisfied Hopkins criteria were not significantly more likely to have a positive result (*p* = 0.78). However, having more than one family member with breast, ovarian, or pancreatic cancer was significantly associated with positive result status (OR 1.46; 95% CI 1.12–1.90; *p* = 0.005), as was having more than one family member with a LS-related cancer (OR 1.33; 95% CI 1.01–1.74; *p* = 0.040). Positive result status was marginally associated with higher Gleason score (OR per unit: 1.17; 95% CI 0.99–1.38; *p* = 0.07), although Gleason information was missing for 49.4% of the cohort. In this cohort, metastatic status was not significantly associated with a positive result (*p* = 0.47); however, this information was only available for 13.1% of the cohort.

**Table 2:** Clinical features associated with positive results^a^.

All gene univariate analysis (*n* = 1812)				
	All gene carriers (*n* = 262)	All gene negative (*n* = 1550)	OR (95% CI)	*p* value
**Percentage**	**14.46%**	**85.54%**		
Age at Dx (IQR)	59 (53.75, 65)	60 (54, 66)	0.99 (0.98, 1.01)	0.32
Personal history of other cancer				
Yes	126	632	1.35 (1.03, 1.75)	**2.67E-02**
No	136	918		
Family history of cancer FDR only^b^				
Yes	222	1295	1.09 (0.77, 1.59)	0.631
No	40	255		
Family history of PC FDR only				
Yes	103	582	1.08 (0.82, 1.41)	0.59
No	159	968		
Family history of PC^c^				
Yes	137	780	1.08 (0.83, 1.41)	0.56
No	125	770		
Met Hopkins FPC criteria				
Yes	46	317	0.92 (0.65, 1.28)	0.61
No	229	1446		
>1 relative with breast, ovarian or pancreatic cancer^c^				
Yes	114	535	1.46 (1.12, 1.9)	**5.14E-03**
No	148	1015		
>1 relative with Lynch-related cancer^c^				
Yes	97	475	1.33 (1.01, 1.74)	**0.04**
No	165	1075		
HBOC criteria				
Yes	67	313	1.36 (1, 1.83)	**1.94E-05**
No	195	1237		
Lynch criteria				
Yes	89	337	1.85 (1.39, 2.45)	**1.94E-05**
No	173	1213		
Metastatic				
Yes	33	164	1.45 (0.57, 4.45)	0.47
No	5	36		
Missing	224	1350	1.19 (0.51, 3.51)	0.71
Gleason score				
<7	17	145		
≥7	106	649	1.39 (0.83, 2.47)	0.23
Missing	139	756	1.57 (0.94, 2.76)	0.099

*CI* confidence interval, *Dx* diagnosis, *FDR* first-degree relative, *FPC* familial prostate cancer, *HBOC* hereditary breast and ovarian cancer, *OR* odds ratio, *PC* prostate cancer.

^a^Clinical variables as reported on test requisition forms.

^b^Nonmelanoma skin cancers and unspecified cancers are excluded.

^c^Includes first, second, and third-degree relatives.

Statistically significant values are in bold.

#### Multivariate analysis

To assess predictors of positive results, a subset of the cohort was analzyed, comprised of men with an available Gleason score who did not have prior genetic testing whose testing included all 14 of these genes (*n* = 524). In a multivariate model, the most informative predictors of a positive result were Gleason score (OR 1.19; 95% CI 0.97–1.45; *p* = 0.096); personal history of breast or pancreatic cancer (OR 3.62; 95% CI 1.37–9.46; *p* = 0.008); >1 family member with breast, ovarian, or pancreatic cancer (OR 2.32; 95% CI 1.48–3.65; *p* = <0.001); and >1 family member with a LS-related cancer (OR 1.97; 95% CI 1.23–3.15; *p* = 0.004) (Table 3). The multivariable adjusted OR for Gleason score represents the comparison per 1 unit increase in Gleason score (i.e., men with a Gleason score of 7 were 19% more likely to test positive than men with a Gleason score of 6). Similarly, men with a personal history of breast or pancreatic cancer were more than three times as likely to test positive than men without these cancers; men with more than one family member with breast, ovarian, or pancreatic cancer were 2.3 times as likely to test positive compared with men without this family history; and men with more than one family member with a LS-related cancer were nearly twice as likely to test positive compared with men who do not have this family history. Ninety-five percent of men with PVs reported at least one of these informative predictors of a positive result.

**Table 3:** Most informative variables for prediction of positive result.

Stepwise coefficients^a,b^			
	Estimate	Pr(>|z|)	OR (95% CI)
Gleason score (numeric)	0.1702	0.096	1.19 (0.97, 1.45)
Personal history: breast or pancreatic cancer	1.2863	0.0082	3.62 (1.37, 9.46)
>1 relative with breast, ovarian, or pancreatic cancer	0.843	2.34E-04	2.32 (1.48, 3.65)
>1 relative with Lynch-related cancer (includes colorectal, endometrial, gastric, ovarian, pancreatic, small bowel, urothelial, kidney, or bile duct cancer)	0.6803	0.0044	1.97 (1.23, 3.15)

^a^The variables presented here are adjusted for all other variables in the table.

^b^Note: “panel tested”, “meet HBOC criteria”, “meet Lynch criteria”, “meet Hopkins FPC criteria” and “metastatic” are not included in any models; all other univariate predictors in Table 2 are included as potential predictors in the selecting procedure: Ashkenazi ethnicity (yes/no); age at prostate cancer (PC); personal history of other cancer; personal history: breast, pancreatic cancer; family history of cancer; family history of cancer first-degree relative (FDR) only; family history of cancer FDR/second-degree relative (SDR); family history of PC; family history of PC FDR only; >1 relative with breast, ovarian, or pancreatic cancer; >1 relative with Lynch-related cancer (includes colorectal, endometrial, gastric, ovarian, pancreatic, small bowel, urothelial, kidney, or bile duct cancer); Gleason score; Gleason score (level 1); Gleason score (level 2), Gleason score (level 3).

## DISCUSSION

The findings from this clinical laboratory genetic testing cohort demonstrate a 9.4–12.1% yield of PVs in men with PC who elected testing and identified predictors of positives result status to include increasing Gleason score; personal history of breast or pancreatic cancer; family history of breast, ovarian, or pancreatic cancer; and family history of LS -associated cancers. The ProstateNext yield of 9.4% was comparable with that previously reported by Pritchard et al. (11.8%) in a 16-gene panel targeted for genes presumed to be associated with PC^1^ in a cohort of men with metastatic PC and in a more recently reported cohort in selected DNA repair genes by Giri et al. (10.9%).^14^ The present yield of other MGPT (12.1%) is lower than recently reported by Nicolosi et al. (17.2%);^15^ however, Nicolosi et al. may have overestimated the PV frequency because the MGPT utilized included up to 80 genes, individuals with prior known familial PVs were not excluded, monoallelic *MUTYH* PVs were included, and the authors included the *CFTR* gene in their data set, which has a variant that is present in 1/24 European Caucasians.^15^ When restricting to genes relevant to PC screening and advanced disease (e.g., *HOXB13* and HRD and MMR genes), findings from this study are similar to Nicolosi et al.

Among PC patients referred for genetic testing, *BRCA2*, *ATM*, *CHEK2*, and *HOXB13* are the most commonly mutated genes. Here, 26/1501 men (1.7%) with no prior genetic testing whose testing included the LS genes (*MLH1*, *MSH2*, *MSH6*, *PMS2*, or *EPCAM*) were found to have a PV in one these MMR genes, and 66/1662 men (4.0%) with no prior testing were found to have *BRCA1* or *BRCA2* PVs. Men with advanced PC who are found to have PVs in the HRD or MMR genes may benefit from targeted therapeutic agents, such as pembrolizumab, platinum therapies, or PARP inhibitors.^7–9^ Positive men with PC may benefit from identifying increased risk for additional cancers, identifying risks to family members, and understanding the cause of their cancer diagnosis.

Stepwise regression analysis was used to identify factors for identifying PC patients with a PV. Factors associated with an increased risk for a positive result include increasing Gleason score; personal history of breast or pancreatic cancer; family history of breast, ovarian, or pancreatic cancer; and family history of LS-associated cancers. These findings are consistent with previous studies and current genetic testing recommendations.^1,10^ Recent expert consensus guidelines reflect that germline testing should be considered in men with aggressive disease, somatic PVs, or family history suggestive of HBOC or LS.^10^ Although a recent publication found no correlation between Gleason score or family history of HBOC or LS-related cancers and positive result status, our present methodology of employing multivariate analysis including only individuals who were tested for all 14 genes included on a prostate gene–specific panel eliminates confounders that were not previously accounted for.^15^ Recently, Nicolosi et al. proposed universal testing among all men with PC, citing an inability to find reliable criteria to predict which men will most benefit from genetic testing.^15^ Although the approach of universal testing proposed by Nicolosi et al. is appealing with respect to increased identification of at-risk patients and simplicity in application, additional studies are needed to assess the diagnostic yield and clinical utility of testing men with clinically localized low-risk disease and no other personal/family history suggestive of inherited cancer predisposition relative to targeted testing strategies.

Despite the potential therapeutic benefit of identifying a PV, we found that on average, there was a six-year delay between time of PC diagnosis and genetic testing. Arguably, targeted therapies for HRD and MMR-deficient tumors have been recently developed and may not have been available for most men at the time of their diagnosis. Going forward, the timing of genetic testing may become more integrated with treatment planning for PC. While the present study focuses on men with PC, ideally genetic testing will identify at-risk men before they are diagnosed so that genetic information can be used for surveillance and clinical decision making. Further analysis of unaffected men is warranted.

There are several limitations that deserve mention, including the fact that this cohort represents men clinically selected for genetic testing; 40.0% of this cohort had multiple primary cancers, indicating high threshold for genetic testing. Further, a clinical laboratory cohort is likely to give an overestimation burden of positive results; therefore, additional studies are needed to determine whether these predictors remain informative in an unselected group of men with PC. Men were tested with one of several multigene panel tests, which could potentially influence the PV frequencies we report, as shown in Table 4. However, our primary analyses limited to individuals tested for the same set of 14 PC susceptibility genes yielded no significant difference in the rate of PVs detected by panel type. This study was also limited by the data provided on the test requisition forms at the time of testing. While results from a recent study of predominately women with a history of breast cancer demonstrate that clinical history reported on test requisition forms is of comparable quality to clinic notes for most probands and their close relatives, it is unknown if the requisition data are similarly valid for prostate cancer and Gleason score reporting.^16^ Additional analysis of the association of metastatic status with positive genetic result status may provide further insight, as metastatic status was available for only 13.1% of the cohort, and we did not find a significant association with positive result status, which differs from previous reports.^1,2^ While the present findings regarding Gleason score were consistent with previous reports, Gleason scores were available for only 51.1% of the cohort.

**Table 4:** Overall results for men with no prior genetic testing/screening.

	ProstateNext				Other MGPT	
*N*	277				1385	
Overall test result (%)						
Positive	26		9.4%		168	12.1%
Inconclusive	44		15.9%		324	23.4%
Moderate risk PV	2		0.7%		13	0.9%
*MUTYH* carrier	0		0.0%		17	1.2%
Negative	205		74.0%		863	62.3%
Gene	PV/VLP	Total # tested	%	PV/VLP	Total # tested	%
*BRCA2*	9	277	3.2%	47	1224	3.8%
*BRCA1*	1	277	0.4%	9	1223	0.7%
*TP53*	1	277	0.4%	4	1367	0.3%
*MLH1*	0	277	0.0%	2	1224	0.2%
*MSH2*	0	277	0.0%	11	1224	0.9%
*MSH6*	0	277	0.0%	5	1224	0.4%
*PMS2*	0	277	0.0%	7	1224	0.6%
*EPCAM*	0	277	0.0%	1	1222	0.1%
*CHEK2*	3	277	1.1%	35	1199	2.9%
*PALB2*	1	277	0.4%	6	1169	0.5%
*ATM*	5	277	1.8%	32	1145	2.8%
*NBN*	2	277	0.7%	2	1057	0.2%
*RAD51D*	0	277	0.0%	0	1031	0.0%
*MUTYH*	0	0	-	23	1151	2.0%
*BRIP1*	0	0	-	1	1037	0.1%
*RAD51C*	0	0	-	1	1038	0.1%
*MRE11A*	0	0	-	2	1031	0.2%
*BARD1*	0	0	-	3	1030	0.3%
*APC*	0	0	-	8	1004	0.8%
*CDKN2A*	0	0	-	5	867	0.6%
*HOXB13*	6	277	2.2%	2	339	0.6%
*SDHD*	0	0	-	1	293	0.3%
*MITF*	0	0	-	3	291	1.0%
*FH*	0	0	-	1	288	0.3%
*FLCN*	0	0	-	1	283	0.4%
*MAX*	0	0	-	1	251	0.4%
*BAP1*	0	0	-	1	244	0.4%
*FANCC*	0	0	-	1	84	1.2%

The present results support multigene panel testing as the primary genetic testing approach for hereditary PC and confirm Gleason score, personal history of breast or pancreatic cancer, and family history of cancers related to HBOC and LS as informative predictors for positive genetic test results. These results provide generalizability of previous findings and support current recommendations for consideration of germline testing in men with PC.

## Supplementary information

Supplementary Information
