# Supplementary material for: Diagnosing hereditary cancer predisposition in men with prostate cancer
Source: Genet Med. 2020 May 22;22(9):1517–23. doi: 10.1038/s41436-020-0830-5 (PMC7462744; doi:10.1038/s41436-020-0830-5)
Supplement: Supplementary file 1 — Supplementary Information [file 41436_2020_830_MOESM1_ESM.docx]

**Table S1: Number of Individuals Tested per Gene (entire cohort)**

| Gene | Total # tested | Gene | Total # tested | Gene | Total # tested | Gene | Total # tested |
| --- | --- | --- | --- | --- | --- | --- | --- |
| *BRCA2* | 1620 | *BRIP1* | 1139 | *SDHD* | 316 | *XRCC2* | 95 |
| *BRCA1* | 1620 | *RAD51C* | 1139 | *VHL* | 316 | *FANCC* | 94 |
| *TP53* | 1794 | *MRE11A* | 1132 | *SDHA* | 315 | *BLM* | 93 |
| *MLH1^a^* | 1636 | *BARD1* | 1130 | *SDHC* | 315 | *GALNT12* | 93 |
| *MSH2^b^* | 1636 | *RAD50* | 1126 | *MITF^i^* | 314 | *RB1* | 77 |
| *MSH6* | 1636 | *APC^e^* | 1118 | *FH* | 311 | *POT1* | 72 |
| *PMS2* | 1636 | *NF1* | 1095 | *FLCN* | 306 | *AIP* | 71 |
| *EPCAM^c^* | 1634 | *BMPR1A* | 1045 | *MET* | 306 | *ALK^j^* | 71 |
| *CHEK2* | 1614 | *SMAD4* | 1045 | *TSC2* | 306 | *CDKN1B* | 71 |
| *PALB2* | 1561 | *CDKN2A* | 945 | *TSC1* | 305 | *NF2* | 71 |
| *ATM* | 1536 | *CDK4* | 873 | *SDHAF2* | 269 | *PHOX2B^k^* | 71 |
| *NBN* | 1445 | *SMARCA4* | 839 | *TMEM127* | 269 | *PRKAR1A* | 71 |
| *PTEN^d^* | 1434 | *POLD1^f^* | 810 | *MAX* | 269 | *PTCH1* | 71 |
| *RAD51D* | 1408 | *POLE^g^* | 810 | *RET* | 268 | *SMARCB1* | 71 |
| *CDH1* | 1359 | *GREM1^h^* | 809 | *BAP1* | 260 | *SMARCE1* | 71 |
| *STK11* | 1280 | *HOXB13* | 662 | *DICER1* | 252 | *SUFU* | 71 |
| *MUTYH* | 1279 | *SDHB* | 316 | *MEN1* | 226 |  |  |

The analytical range of each gene includes all coding domains and well into the flanking 5’ and 3’ ends of all the introns and untranslated regions, with the following additions/exceptions: ^a,b,d,e^sequencing of the promoter regions were analyzed and reported for the following: *MLH1* (c.-337 to c.-194), *MSH2* (c.-318 to c.-65); *PTEN* ( c.-1300 to c.-745), all promoter 1B gross deletions as well as single nucleotide substitutions within the promoter 1B YY1 binding motif of *APC* (NM_001127511 c.-196_-186); ^c^only gross deletions encompassing the 3’ region of *EPCAM* were reported; ^f,g^ missense variants located outside of the exonuclease domains of *POLD1* and *POLE* (codons 311-541 and 269-485, respectively) were not reported; ^h^ only the status of the 40kb 5’UTR gross duplication in *GREM1* was analyzed and reported; ^i^only the status of the *MITF* c.952 (p.E318K) alteration was analyzed and reported; ^j^ only variants located within the kinase domain of *ALK* (c.3286-c.4149) were reported; ^k^ the polyalanine region of *PHOX2B* is excluded from analysis

**Table S2: Positive Rate by Panel and Prior Testing Status**

|  | ProstateNext Prior GT | % | ProstateNext No Prior GT | % | Other Panel Prior GT | % | Other Panel No Prior GT | % | All | % |
| --- | --- | --- | --- | --- | --- | --- | --- | --- | --- | --- |
| N | 7 | 0.3% | 277 | 12.9% | 143 | 6.7% | 1385 | 76.4% | 1812 | 100.0% |
| Overall Test Result (%)^a^ |  |  |  |  |  |  |  |  |  |  |
| Positive | 4 | 57.1% | 26 | 9.4% | 30 | 21.0% | 168 | 12.1% | 228 | 12.6% |
| Inconclusive | 1 | 14.3% | 44 | 15.9% | 36 | 25.2% | 324 | 23.4% | 405 | 22.4% |
| Moderate risk mutation | 0 | 0.0% | 2 | 0.7% | 1 | 0.7% | 13 | 0.9% | 16 | 0.9% |
| *MUTYH* carrier | 0 | 0.0% | 0 | 0.0% | 1 | 0.7% | 17 | 1.2% | 18 | 1.0% |
| Negative | 2 | 28.6% | 205 | 74.0% | 75 | 52.4% | 863 | 62.3% | 1145 | 63.2% |

**Table S3: Positive Rate by Gene in Men with no Prior Genetic Testing (entire cohort)**

| Gene  (Prostate Specific) | N. Positive | N. Tested | Mutation Rate | Gene  (non-Prostate Specific) | | N. Positive | N Tested | Mutation Rate |
| --- | --- | --- | --- | --- | --- | --- | --- | --- |
| *BRCA2* | 56 | 1591 | 3.7% | *MUTYH* | 23 | | 1151 | 2.0% |
| *CHEK2* | 38 | 1476 | 2.6% | *FANCC* | 1 | | 84 | 1.2% |
| *ATM* | 37 | 1422 | 2.6% | *MITF* | 3 | | 291 | 1.0% |
| *HOXB13* | 8 | 616 | 1.3% | *APC* | 8 | | 1004 | 0.8% |
| *BRCA1* | 10 | 1500 | 0.7% | *CDKN2A* | 5 | | 867 | 0.6% |
| *MSH2* | 11 | 1501 | 0.7% | *FLCN* | 1 | | 283 | 0.4% |
| *PMS2* | 7 | 1501 | 0.5% | *BAP1* | 1 | | 244 | 0.4% |
| *PALB2* | 7 | 1446 | 0.5% | *NBN* | 4 | | 1334 | 0.3% |
| *TP53* | 5 | 1644 | 0.3% | *BARD1* | 3 | | 1030 | 0.3% |
| *MSH6* | 5 | 1501 | 0.3% | *SDHD* | 1 | | 293 | 0.3% |
| *MLH1* | 2 | 1501 | 0.1% | *FH* | 1 | | 288 | 0.3% |
| *EPCAM* | 1 | 1499 | 0.1% | *MRE11A* | 2 | | 1031 | 0.2% |
|  |  |  |  | *RAD51C* | 1 | | 1038 | 0.1% |
|  |  |  |  | *BRIP1* | 1 | | 1037 | 0.1% |
|  |  |  |  |  |  | |  |  |
|  |  |  |  |  |  | |  |  |
|  |  |  |  |  |  | |  |  |
|  |  |  |  |  |  | |  |  |
|  |  |  |  |  |  | |  |  |

**Table S4: Clinical History in Men with Pathogenic Variants not associated with Prostate Cancer**

**Gene Classification Clinical Cancer History***APC*  PV polyposis, basal cell carcinoma (56), prostate (59)
*BAP1* PV mesothelioma (55), basal cell carcinoma (41), kidney (51), brain tumor (56), prostate (51)

*CDKN2A* PV melanoma (34), prostate (63) *CDKN2A* PV melanoma (63,66), thyroid (63), prostate (68)
*CDKN2A* PV melanoma (55, 63), prostate (49)
MAX PV pheochromocytoma (52), kidney (68), prostate (52) *MITF /SDHD* PV colorectal ca (73), paraganglioma (77), prostate (74)
*MITF* PV melanoma (73), prostate (58)
*MITF* PV bladder (66), kidney (63), prostate (63)

Table S5: Clinical History in Men with Multiple Pathogenic Variants

**Pathogenic Variants Identified Personal Clinical Cancer History***ATM*/*BRCA1* kidney (70), prostate (73), gastric (74)
*MITF*/*SDHD* colorectal (73), prostate (74), paraganglioma (77)
*ATM*/*BRCA2* prostate (55), male breast (70)
*CHEK2*/*MSH2* colorectal (50, 51), prostate (68), pancreatic (72)
*MSH2*/*PMS2* colorectal (35), prostate (69), small intestine (70)
*ATM*/*MRE11A* prostate (57), leukemia (58), melanoma (60,62)
BRCA1/MSH6 prostate (46), colorectal (53), kidney (56)
*ATM*/*FH* prostate (58)
*PMS2*/*CHEK2* pancreatic (34), lymphoma (34), prostate (35)
*BRCA2*/*CHEK2* prostate (61)
*ATM*/*BRCA2* melanoma (59), prostate (69)
*MRE11A*/*CHEK2*  prostate (46)
*CHEK2*/*ATM* prostate (63), kidney (63), gastric (64)
*BRCA2*/*PMS2* prostate (61), biliary tract (63)

**Figure S1: Positive Results in Individuals with Prior Genetic Testing**
